# Circulating T‐cell subsets discrepancy between bipolar disorder and major depressive disorder during mood episodes: A naturalistic, retrospective study of 1015 cases

**DOI:** 10.1111/cns.14361

**Published:** 2023-07-25

**Authors:** Shaoli Li, Duo Lv, Chao Qian, Jiajun Jiang, Peifen Zhang, Caixi Xi, Lingling Wu, Xingle Gao, Yaoyang Fu, Danhua Zhang, Yiqing Chen, Huimin Huang, Yiyi Zhu, Xiaorong Wang, Jianbo Lai, Shaohua Hu

**Affiliations:** ^1^ Department of Psychiatry, The First Affiliated Hospital Zhejiang University School of Medicine Hangzhou China; ^2^ The Key Laboratory of Mental Disorder's Management in Zhejiang Province Hangzhou China; ^3^ Department of Medical Oncology, The Second Affiliated Hospital Zhejiang University School of Medicine Hangzhou China; ^4^ Zhejiang Engineering Center for Mathematical Mental Health Hangzhou China; ^5^ Department of Clinical Pharmacy, The First Affiliated Hospital Zhejiang University School of Medicine Hangzhou China; ^6^ Shaoxing 7th People's Hospital Shaoxing China; ^7^ Wenzhou Medical University Wenzhou China; ^8^ Department of Neurobiology, NHC and CAMS Key Laboratory of Medical Neurobiology, School of Brain Science and Brian Medicine, MOE Frontier Science Center for Brain Science and Brain‐Machine Integration Zhejiang University School of Medicine Hangzhou China

**Keywords:** biomarker, bipolar disorder, inflammation, major depressive disorder, T cell

## Abstract

**Aims:**

We aimed to investigate whether peripheral T‐cell subsets could be a biomarker to distinguish major depressive disorder (MDD) and bipolar disorder (BD).

**Methods:**

Medical records of hospitalized patients in the Department of Psychiatry, the First Affiliated Hospital, Zhejiang University School of Medicine, from January 2015 to September 2020 with a discharge diagnosis of MDD or BD were reviewed. Patients who underwent peripheral blood examination of T‐cell subtype proportions, including CD3+, CD4+, CD8+ T‐cell, and natural killer (NK) cells, were enrolled. The Chi‐square test, *t*‐test, or one‐way analysis of variance were used to analyze group differences. Demographic profiles and T‐cell data were used to construct a random forest classifier‐based diagnostic model.

**Results:**

Totally, 98 cases of BD mania, 459 cases of BD depression (BD‐D), and 458 cases of MDD were included. There were significant differences in the proportions of CD3+, CD4+, CD8+ T‐cell, and NK cells among the three groups. Compared with MDD, the BD‐D group showed higher CD8+ but lower CD4+ T‐cell and a significantly lower ratio of CD4+ and CD8+ proportions. The random forest model achieved an area under the curve of 0.77 (95% confidence interval: 0.71–0.83) to distinguish BD‐D from MDD patients.

**Conclusion:**

These findings imply that BD and MDD patients may harbor different T‐cell inflammatory patterns, which could be a potential diagnostic biomarker for mood disorders.

## Graphical abstract

Peripheral blood pattern of T‐cell subsets was significantly different between patients with bipolar disorder and those with major depressive disorder. The random forest model derived from data on serum T‐cell subsets and demographic profiles can effectively distinguish bipolar depression and unipolar depression.

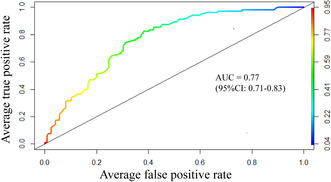

## 1 INTRODUCTION

The disease burden of mood disorders, predominantly including bipolar disorder (BD) and major depressive disorder (MDD), is greatly heavy worldwide. BD ranks as the 17th most disabling disease in the world,^1^ and the lifetime prevalence and 12‐month prevalence of BD were, respectively, 2.4% and 1.5%, as reported by the World Mental Health Survey Initiative.^2^ Moreover, as a chronic and disabling disease, MDD affected over 350 million people globally and ranked second in terms of disease burden.^3^ According to the 5th edition of the Diagnostic and Statistical Manual of Mental disorders (*DSM‐5*), clinical diagnosis of BD and MDD is mainly symptom‐driven and lacks objective biomarkers.^4^ However, the majority of patients with BD suffer from depressive episodes mostly rather than manic/hypomanic episodes and may manifest with psychotic symptoms across the disease course. Therefore, the clinical misdiagnosis rate of BD is extremely high. A study showed that more than one‐third of patients sought professional help within 1 year after the first onset of symptoms, but unfortunately, 69% were misdiagnosed, most commonly being diagnosed with depression.^5^ Strikingly, it might cost an average of 8 or more years to get a confirmed diagnosis of BD.^5^ Another study showed that 15% of MDD patients receiving treatment in primary care facilities might have undiagnosed BD.^6^ Based on these facts, objective biological markers are urgently needed to help distinguish BD from MDD.

The general pattern of mature T lymphocytes, namely CD3+ T‐cell, represents the immune function state of the human body and includes predominantly CD4+ and CD8+ T‐cell subsets.^7^ As the inducible T cells, CD4+ T cells are the most important helper cells in regulating the immune response and can be classified into T helper 1, 2, 17 (Th1, Th2, and Th17), and T regulatory (Treg) cells.^8^ CD8+ T cells are inhibitory T lymphocytes, directly killing cells in the immune response. The ratio of CD4+ and CD8+ is a sensitive index for clinical diagnosis of human immune function, and it is also regarded as an indicator of immunosenescence.^9^ Natural killer (NK) cells are known as the first responder in the immune response to infected or cancerous cells. In recent years, the role of immune activation in the pathogenesis of MDD and BD has been gradually recognized. Early studies have found disturbances in the circulating counts and function of immune cells in MDD and BD by examining the various cells of the lymphoid lineage, which consists primarily of T cells, B cells, and NK cells. Lately, some studies focused on investigating the role of T cells and NK cells in mood disorders.^10^, ^11^, ^12^ A recent systematic review suggested that in patients with MDD, Treg cell deficits were associated with inflammatory monocyte activation.^13^ Furthermore, another study also identified CD4+ Th cell deficits in patients with MDD.^10^ In patients with BD, however, data regarding immune cell subsets are scarce and controversial. One study showed an enhancement of pro‐inflammatory monocyte and anti‐inflammatory T‐cell forces in BD patients,^14^ while another study reported that compared to healthy controls, BD patients presented an increased circulating proportion of monocytes (CD14+) and a lower proportion of T‐cell (CD3+) and cytotoxic T cells (CD3 + CD8+),^15^ which was consistent with our previous findings.^16^ Moreover, BD patients showed an increased proportion of activated T cells (CD4+CD25+) and a lower proportion of soluble interleukin (IL)‐10 expressing Treg cells (CD4+CD25+FoxP3+IL10+), suggesting increased activation of monocytes and lymphocytes.^15^ Another study found a decreased percentage of CD4+ lymphocytes that expressed chemokine receptors in BD patients.^17^ Taken together, these findings suggested that T‐cell subsets and related immune disturbance might underline the pathogenesis of BD and MDD. However, the discrepancy in the immune activation between BD and MDD remains largely unexplored. Therefore, investigating T‐cell subsets differences between these two mood disorders may provide insight into the pathophysiological underpinnings and aid in precise diagnosis.

To date, several studies have explored the difference in T‐cell subsets between BD and MDD, but with small sample sizes and conflicting results. Wu Wei et al.^16^ found that compared with MDD, BD patients with a depressive episode showed lower CD8+ T‐cell, while there was no difference in the CD4+ T‐cell between the two groups. However, another study showed that the circulating levels of CD4+ T cells were higher in BD patients as compared to the MDD.^18^ Herein, we aimed to increase the sample size to investigate whether peripheral blood proportions of various T‐cell subsets were different during mood episodes of MDD and BD. Given the clinical need for differential diagnosis of bipolar depression and unipolar depression, we further constructed a random forest model based on peripheral T‐cell data to verify its efficacy as a diagnostic biomarker for mood disorders.

## 2 METHODS

This retrospective study enrolled hospitalized patients from the Department of Psychiatry, the First Affiliated Hospital, Zhejiang University School of Medicine from January 2015 to September 2020. Due to the anonymous, retrospective, and nonintervention nature of this study, informed consent from all patients was waived, and this study was approved by the Hospital Ethical Committee (Approval number: 2021‐IIT‐513).

### 2.1 Study subjects

With the assistance of the Electronic Case Record System, patients with a discharge diagnosis of BD or MDD were successively screened. The diagnosis was made according to *DSM‐5*. Subjects who met the following criteria were included: (1) no comorbid severe physical diseases, such as autoimmune diseases and hematological system diseases; (2) no history of infection, fever, or diarrhea at least three months before admission; (3) no use of medications that were known to have an immunoregulatory function; (4) no comorbidity with any other psychiatric disorders; and (5) malnutrition with a body mass index less than 17.5 kg/m^2^.

### 2.2 Data collection

For patients enrolled, the demographic and clinical profiles, including gender, age, education level, marital status, obstetrical history, course of illness, history of psychotropic medications, and final diagnosis on discharge, were collected. According to the diagnosis, all patients were divided into three groups: BD‐M (bipolar disorder and manic episode), BD‐D (bipolar disorder and depressive episode), and MDD. The peripheral proportion of different T‐cell subsets, including CD3+, CD4+, CD8+ T cells, and NK cells, was a routine examination following hospitalization in the Department of Psychiatry, the First Affiliated Hospital, Zhejiang University School of Medicine. Venous blood samples were collected at 7 a.m. in a fasting state from all individuals. Flow cytometry was used to determine T‐cell subsets proportion (see Table S1).^19^ In this study, the existing results of the T‐cell subsets proportion of all subjects were directly extracted from the Electronic Case Record System.

### 2.3 Statistical analysis

All the statistical analysis was performed with SPSS 19.0 for Windows (SPSS, Inc.). The categorical variables were presented as numbers (frequency) and compared with the Chi‐square test. The continuous variables were tested for normality by Shapiro–Wilk test. When variables conformed to normal distribution, they were presented as mean ± standard deviations (SD) and compared with one‐way analysis of variance and *t*‐test. When data did not exhibit a normal distribution, they were presented as median and interquartile range and analyzed via a nonparametric equivalent. The statistical significance was set at *p* < 0.05, and all tests were two‐tailed. Bivariate analysis was used to investigate the relationship between T‐cell subsets and age. To further verify the diagnostic role of peripheral T‐cell profiles for distinguishing BD‐D and MDD patients, a machine learning called random forest analysis was performed using R software (version 4.0.4). Being a type of pattern recognition, machine learning represents a range of computational techniques used to analyze complex data by identifying patterns of interaction among several variables.^20^ Moreover, Wollenhaupt et al.^14^ previously constructed a random forest model combined with peripheral biomarker measurements (IL‐4, thiobarbituric acid reactive substances, and IL‐10) to help differentiate patients with BD from those with MDD. Based on the above analysis, nine significantly different features across groups (age, education year, marital status, delivery history, CD3+, CD4+, CD8+ T‐cell, the ratio of CD4+ and CD8+ proportion, and NK cells) were extracted to construct a random forest model. We used a split validation method, and 75% of the samples were randomly selected as the training set, and the remaining as the test set. The random algorithm was used to establish the training model in the training set, and the test set was used for prediction. As the random forest is a black‐box algorithm, we ranked the importance of each predictor using the mean decreased Gini (MDG) index. MDG index measures the contribution of each variable to the homogeneity of the model. The higher the MDG index of the variable, the more significant it is to distinguish BD‐D from MDD.^21^ Finally, we used the area under the receiver operating characteristics (ROC) curve (AUC) to evaluate the model performance.

## 3 RESULTS

### 3.1 Demographic and clinical characteristics

Totally, 98 cases of BD‐M, 459 cases of BD‐D, and 458 cases of MDD met the inclusion criteria and were included in the final analysis. Among the BD‐M, BD‐D, and MDD groups, no difference in gender was reported. Due to the naturalistic feature of this study, however, the age of patients in the three groups was significantly different (*p* < 0.001), and those in the BD‐D group (23.9 ± 12.5‐year‐old) were the youngest. Also, education level, marital status, and delivery history were different across the three groups (all *p* < 0.001). Patients in the BD‐M group were averagely the most educated (12.3 ± 3.9 years). In addition, there was no significant difference in illness duration (*p* = 0.070) or history of psychotropic medications (*p* = 0.205) across the three groups. Details on demographic and clinical profiles are shown in Table 1.

**TABLE 1:** Demographic and clinical profiles of all subjects across three groups.

	BD‐M *N* = 98	BD‐D *N* = 459	MDD *N* = 458	Total *N* = 1015	*p* Value
Age, years	30.5 ± 13.3	23.9 ± 12.5^a^	40.4 ± 18.2^a^ ^,^ ^b^	32.0 ± 17.3	<0.001
Education, years	12.3 ± 3.9	10.7 ± 3.1^a^	10.0 ± 4.2^a^	10.5 ± 3.8	<0.001
*Gender*					0.305
Male	28 (28.6%)	134 (29.2%)	114 (24.9%)	276 (27.2%)	
Female	70 (71.4%)	325 (70.8%)	344 (75.1%)	739 (72.8%)	
*Marriage*					<0.001
Unmarried	50 (51.0%)	352 (76.7%)^a^	153 (33.4%)^a^ ^,^ ^b^	555 (54.7%)	
Married	48 (49.0%)	107 (23.3%)^a^	305 (66.6%)^a^ ^,^ ^b^	460 (45.3%)	
*Delivery*					<0.001
No	59 (60.2%)	356 (77.6%)^a^	155 (33.8%)^a^ ^,^ ^b^	570 (56.2%)	
Yes	39 (39.8%)	103 (22.4%)^a^	303 (66.2%)^a^ ^,^ ^b^	445 (43.8%)	
Illness duration, year	4.9 ± 5.2	4.2 ± 5.1	5.0 ± 6.7^b^	4.6 ± 5.9	0.070
*History of psychotropic medications*					
No	25 (25.5%)	97 (21.1%)^a^	119 (26.0%)^a^ ^,^ ^b^	233 (23.0%)	
Yes	73 (74.5%)	362 (78.9%)^a^	339 (74.0%)^a^ ^,^ ^b^	782 (77.0%)	0.205

*Note*: Chi‐square test was used to compare the categorical variables across three groups. For continuous variables, we used one‐way analysis of variance to compare the difference among three groups. When *p* < 0.05, we did a post hoc multiple comparisons using Bonferroni test if the data were homoscedasticity, or Dunnett's test was used. All the *p* values in the table were uncorrected values.

^a^ Compared to BD‐M, the difference was significant.

^b^ Compared to BD‐D, the difference was significant.

### 3.2 Different proportions of T‐cell subsets in MDD, BD‐D, and BD‐M

**FIGURE 1:**
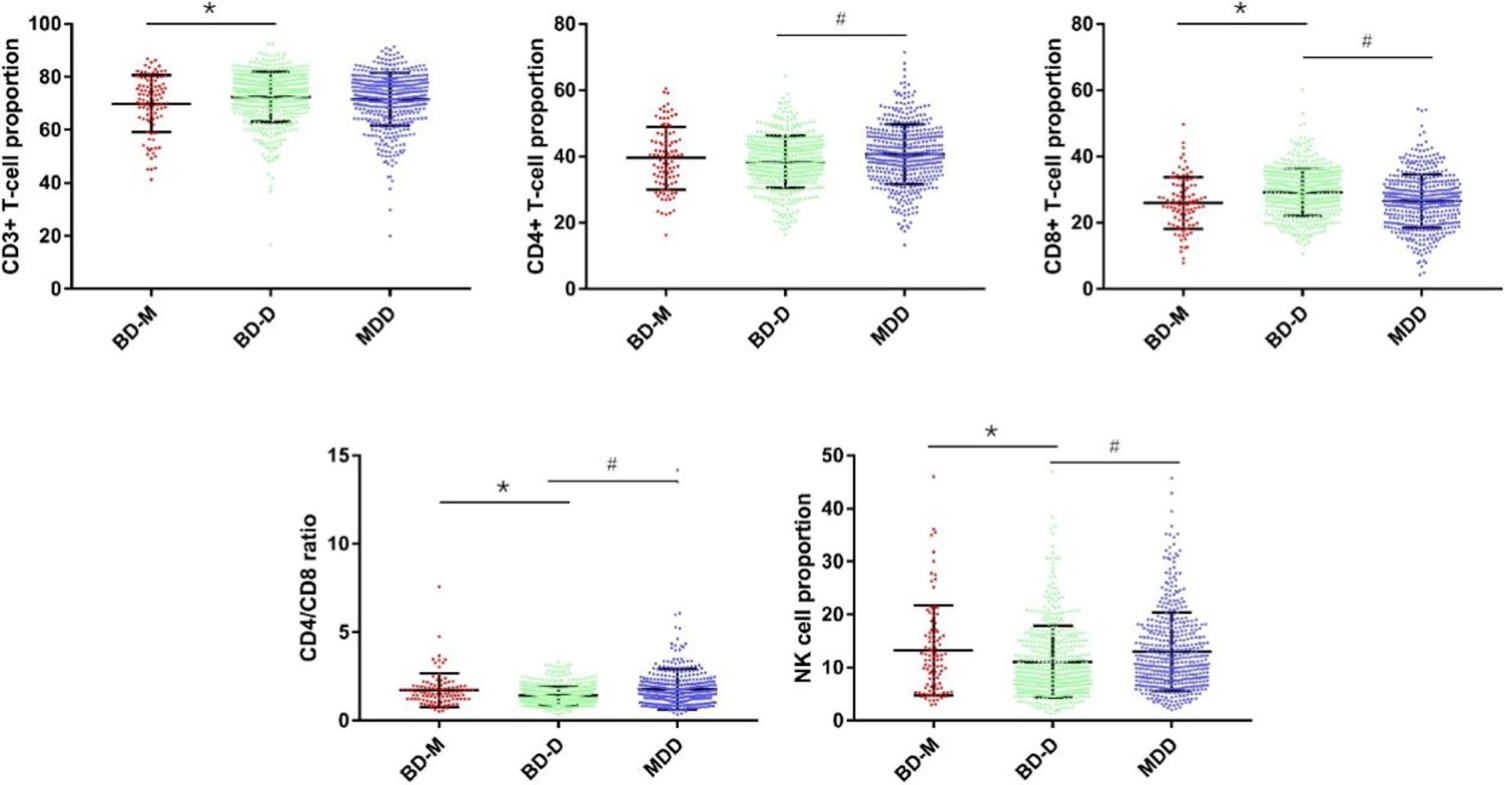
Comparison of peripheral T‐cell proportions and NK cells across three groups. Figure showed the representative dot plot of CD3+, CD4+, CD8+ T‐cell, CD4+/CD8+ ratio, and NK cells. BD‐D, bipolar disorder depressive episode; BD‐M, bipolar disorder manic episode; NK: natural killer. *Compared to BD‐M, *p* < 0.05; ^#^Compared to BD‐D, *p* < 0.05. *p* < 0.05, representing a statistically significant difference between groups.

Peripheral proportions of different T‐cell subsets, including CD3+, CD4+, CD8+ T‐cell, and NK cells, as well as the CD4+ and CD8+ ratio, were all different across the three groups (all *p* < 0.05). Of note, the BD‐D group reported the lowest CD4+/CD8+ ratio, while the BD‐M and MDD groups did not show any differences in the CD4+/CD8+ ratio. Compared to those in the BD‐M group, patients in the BD‐D group showed higher proportions of CD8+ T‐cell, but lower levels of CD4+/CD8+ ratio and NK cells. Compared with the MDD group, the BD‐D group showed a higher proportion of CD8+ T‐cell and lower levels of CD4+ T‐cell, NK cells, and CD4+/CD8+ ratio (see Table 2 and Figure 1). Considering the differences in age among the three groups, we used bivariate analysis to analyze the relationship between T‐cell subsets and age. The result showed a significant relationship between age and the T‐cell subsets (CD4+, CD8+ T‐cell, CD4+/CD8+ ratio), but the Pearson correlation coefficient was low (all *R* < 0.5), suggesting that the correlation between age and T‐cell subsets was relatively weak (see Table S1).

**TABLE 2:** Comparison of peripheral T‐cell proportions across three groups.

	BD‐M *N* = 98	BD‐D *N* = 459	MDD *N* = 458	Total *N* = 1015	*F* value	*p* Value
CD3+ (%)	69.92 ± 10.75	72.48 ± 9.46	71.50 ± 9.94	71.8 ± 9.82	3.12	0.044
CD4+ (%)	39.53 ± 9.48	38.39 ± 7.82	40.56 ± 9.03^b^	39.53 ± 8.66	7.38	0.001
CD8+ (%)	25.92 ± 7.76	29.2 ± 7.12^a^	26.65 ± 7.92^b^	27.69 ± 7.72	16.18	<0.001
CD4+/CD8+	1.73 ± 0.95	1.42 ± 0.53^a^	1.72 ± 0.82^b^	1.61 ± 0.91	22.36	<0.001
NK (%)	13.23 ± 8.48	11.09 ± 6.75^a^	13.00 ± 7.42^b^	12.17 ± 7.29	9.20	<0.001

*Note*: The one‐way analysis of variance was used to compare the peripheral T‐cell proportions across three groups. When *p* < 0.05, we did a post hoc multiple comparisons using Bonferroni test if the data were homoscedasticity, or Dunnett's test was used.

Abbreviation: NK, natural killer.

^a^ Compared to BD‐M, the difference was significant.

^b^ Compared to BD‐D, the difference was significant.

### 3.3 Random forest model distinguished BD‐D from MDD

Totally 687 training samples were used to establish the random forest model, and 230 test samples were used to validate the model. The demographic and clinical variables of the training and testing set are presented in Tables 3 and 4, respectively. There were no significant differences in the demographic and clinical variables between the BD‐D and MDD groups (all *p* > 0.05).

**TABLE 3:** Demographic and clinical profiles of the training set and the test set.

	Training set *N* = 687	Test set *N* = 230	Total *N* = 917	*p* Value
Age, years	31.9 ± 17.6	32,7 ± 17.9	32.1 ± 17.7	0.541
Education, years	10.4 ± 3.6	10.1 ± 3.9	10.3 ± 3.7	0.318
*Gender*				0.973
Male	183 (26.6%)	61 (26.5%)	244 (26.6%)	
Female	504 (73.4%)	169 (73.5%)	673 (73.4%)	
*Marriage*				0.919
Unmarried	379 (55.2%)	126 (54.8%)	505 (55.1%)	
Married	308 (44.8%)	104 (45.2%)	412 (44.9%)	
*Delivery*				0.740
No	385 (56.0%)	126 (54.8%)	511 (55.7%)	
Yes	302 (44.0%)	104 (45.2%)	406 (44.3%)	
Illness duration, year	4.6 ± 6.0	4.7 ± 6.1	4.6 ± 6.0	0.762
*History of psychotropic medications*				0.743
No	160 (23.3%)	56 (24.3%)	216 (23.6%)	
Yes	527 (76.7%)	174 (75.7%)	701 (76.4%)	

*Note*: Chi‐square test was used to compare the categorical variables between training set and test set. For continuous variables, *t*‐test was used to compare the difference between two groups.

**TABLE 4:** Comparison of peripheral T‐cell proportions between the training and the test set.

	Training set *N* = 687	Test set *N* = 230	Total *N* = 917	*p* Value
CD3+	72.00 ± 9.59	72.00 ± 10.08	72.0 ± 9.71	0.957
CD4+	39.17 ± 8.33	40.39 ± 8.98	39.48 ± 8.51	0.061
CD8+	28.05 ± 7.72	27.54 ± 7.36	27.93 ± 7.63	0.378
CD4+/CD8+	1.55 ± 0.69	1.62 ± 0.75	1.57 ± 0.71	0.162
NK	12.22 ± 7.33	11.52 ± 6.57	12.05 ± 7.15	0.198

*Note*: *t*‐Test was used to compare the difference between training set and test set.

**FIGURE 2:**
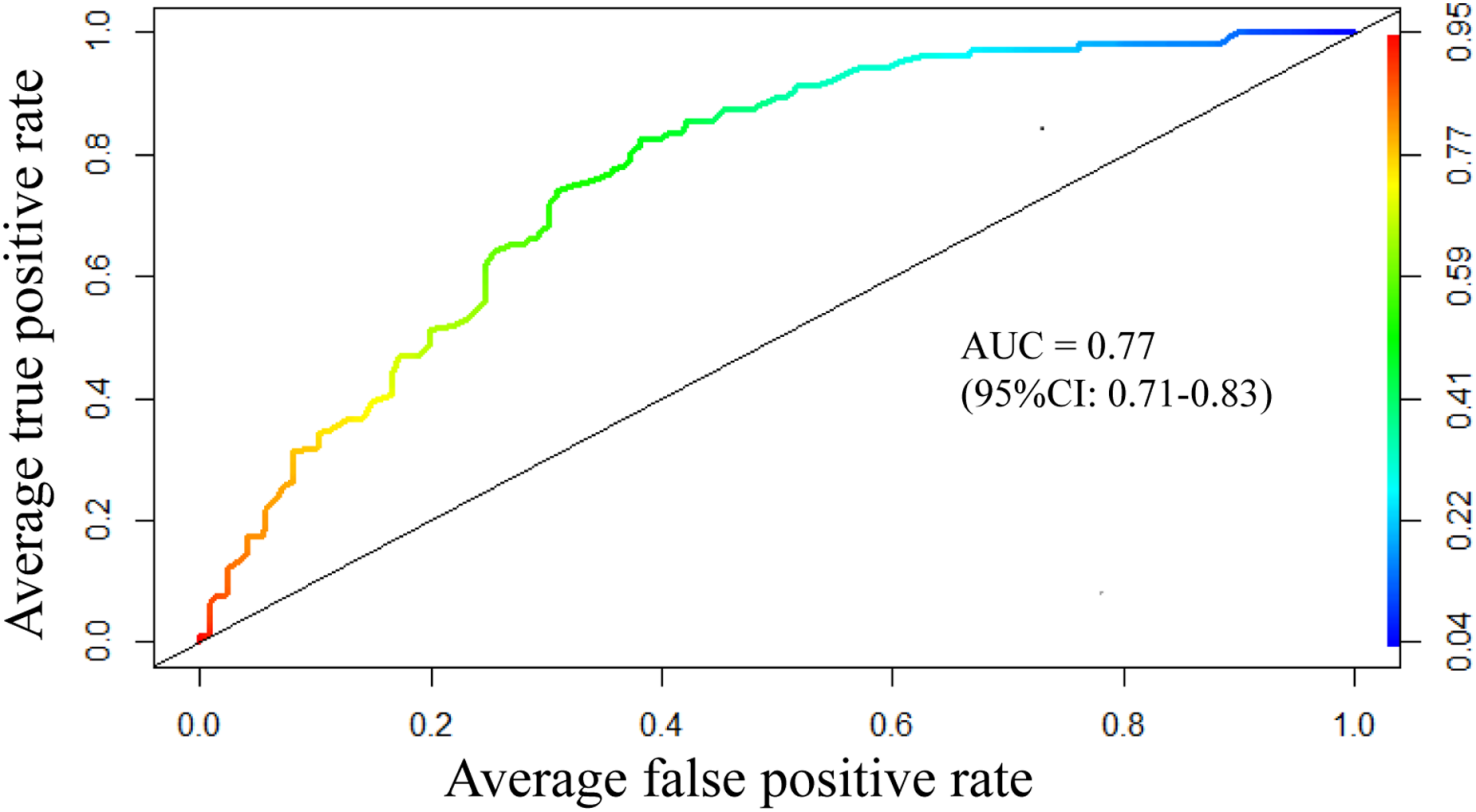
The receiver operating characteristic curve in the random forest sample. AUC = 0.77, 95% confidence interval (95%CI), 0.71–0.83. The vertical axis on the right represented the prediction probability of the test sample estimated by the random forest model. According to the prediction probability and true value of the test sample, the receiver operating characteristic curve was drawn. AUC, area under the curve; between 0.5 and 1. An evaluation index to measure the performance of a binary classification model. When AUC >0.7, the model is considered reliable. 95%CI, 95% confidence interval.

**FIGURE 3:**
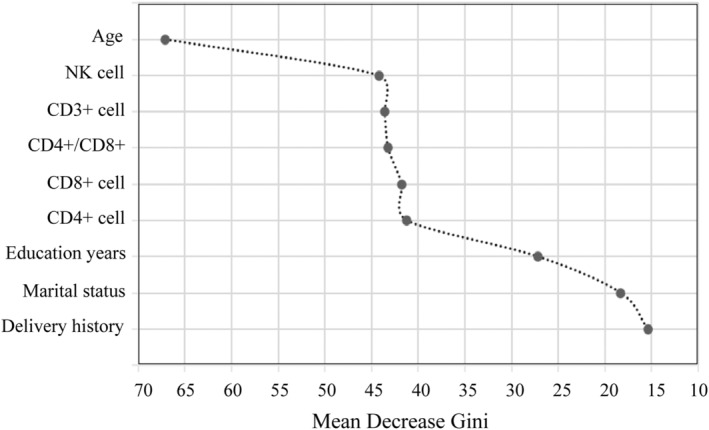
Variable importance in distinguishing patients with bipolar depression and major depressive disorder estimated by the random forest model. The horizontal axis represented mean decrease in Gini Impurity Index (a weighted average of reduction in leaf node impurities), and the vertical axis represented the included predictor. The more the MDG index, the more significant the variable, and predictors were presented from top to bottom in order of importance. MDG, mean decreased Gini.

The error rate for predicting BD‐D and MDD patients was 26.77% and 34.34%. The Out‐of‐Bag (OOB) estimate of the error rate was 30.42%. The AUC used to evaluate the prediction ability of the model was 0.77 (95%CI: 0.71–0.83; see Figure 2). According to the MDG index (see Figure 3) of the nine variables, the strongest predictions between BD‐D and MDD was age, followed by NK cells, CD3+ T cells, the ratio of CD4+ and CD8+ T cells, CD8+ T cells, and CD4+ T cells. Of note, the ratio of CD4+ and CD8+ T cells played an important role in distinguishing the BD‐D and MDD, which was consistent with our findings.

## 4 DISCUSSION

In the current study, we compared the proportion of peripheral blood T‐cell subsets of 1015 patients and found that peripheral proportions of different T‐cell subsets, including CD3+, CD4+, CD8+ T‐cell, and NK cells, as well as the CD4+/CD8+ ratio, were all different across the three groups. Compared with MDD, BD‐D patients presented lower CD4+ T‐cell and NK cells but higher CD8+ T‐cell proportions. Of note, the ratio of CD4+ and CD8+ proportion was significantly lower in BD‐D than that in the MDD group. These findings indicated that BD‐D and MDD subjects displayed a significantly different T‐cell immune profile, which might be helpful for the differential diagnosis between these two mood disorders.

In our study, compared with MDD, BD‐D patients presented lower CD4+ but higher CD8+ T‐cell. However, previous research showed there was no significant difference in CD4+ T‐cell between the two groups, and the proportion of CD8+ T‐cell was also lower in BD‐D patients than that in MDD.^16^ In addition, another study presented that BD had a higher level of CD4+ T‐cell than MDD patients.^18^ Though the results of different studies were conflicting, they all suggested that there were differences in T‐cell immune regulation between patients with BD and MDD. As potentially chronic and non‐infectious inflammatory diseases, BD and MDD were thought to be associated with abnormal levels of circulating immune cells and regulatory cytokines.^12^, ^22^, ^23^, ^24^ Several studies have shown that T cells were involved in the pathogenesis of mood disorders, and the interactions between mood, behavior, and the immune system were mediated by the endocrine and nervous systems.^12^, ^25^, ^26^, ^27^ Most patients with mood disorders were affected by chronic stress, resulting in prolonged hypothalamic–pituitary–adrenal (HPA) activation, which played a crucial role in regulating the immune axis by releasing glucocorticoids targeting immune organs and cells.^28^, ^29^, ^30^, ^31^ Overall, chronic stress might suppress adaptive immunity through the HPA axis, ultimately affecting the proliferation and differentiation of T‐cell. This may be one possible explanation for the discrepancy in T‐cell subsets observed between the BD and MDD groups in our study. Repeated exposure to psychological stress may facilitate T‐cell trafficking to the brain, which subsequently lead to neuroinflammation and predispose the onset of mood disorders.^32^, ^33^ A recent postmortem study reported an increased T‐cell infiltration in the brain parenchyma in patients with mood disorders,^34^ further suggesting an association among mood disorders, neuroinflammation, and T cells. Several studies have suggested that T cells might promote neuroinflammation in mood disorders via the activation of microglia and astrocytes,^25^, ^33^, ^35^ and in vitro, Th1 and Th17 cells could potentiate microglia activation, thus shifting the balance toward an inflammatory M1‐like phenotype, which was related to the onset and development of major psychiatric disorders.^36^ Activated microglia were thought to modulate key stress‐responsive brain regions, including the hypothalamus, prefrontal cortex, hippocampus, and amygdala.^37^ Huang et al.^38^ found a significant relationship between the CD4+/CD8+ ratio and the proliferation rate of the hippocampal dentate gyrus, which may contribute to brain dysfunction and mood dysregulation. These findings help to explain the difference in the CD4+/CD8+ ratio between BD‐D and MDD in our study. Moreover, previous studies have indicated that patients with mood disorders might harbor abnormal levels of regulatory cytokines.^39^, ^40^, ^41^ A meta‐analysis also confirmed that the IL‐2 receptor, IL‐6, IL‐10, IL‐13, IL‐18, IL‐12, and tumor necrosis factor‐alpha levels were increased in MDD patients.^40^ Do Prado and colleagues showed that multiple Th1/Th2/Th17 cytokines, including IL‐2, IL‐4, IL‐6, IL‐10, IL‐17, interferon γ (INF‐γ), and tumor necrosis factor‐alpha, were significantly increased in BD patients.^39^ The abnormal cytokine levels in patients with mood disorders implied changes in circulating immune cells. In addition, it seems that human Treg cells are relatively unstable and may differentiate into Th17 producer cells under specific conditions.^42^ Mounting evidence also showed that patients with mood disorders had an increased amount of Th17 cells, as well as elevated plasma IL‐17 levels.^35^, ^39^, ^43^, ^44^ The increased Th17 might activate microglia and astrocytes, resulting in higher susceptibility to depressive behaviors.^45^ In our study, T‐cell subsets were significantly different between BD and MDD patients, suggesting that immune cells are involved in the occurrence and development of mood disorders. The discrepancy of T‐cell subsets may result from complex interactions among genetic, epigenetic, and environmental factors.^46^

Our study showed higher NK cells in MDD in comparison to BD‐D patients. As innate immune cells, NK cells are capable of dampening down the activation of microglial cells.^47^ Raised numbers of NK cells were considered to suppress activated microglia, leading to mood disorders.^48^ This suggested that the levels of NK cells might influence the inflammatory status of the central nervous system, which in turn caused different types/episodes of mood disorders. In addition, Tarantino et al.^49^ observed a specific impairment of IFN‐γ production in NK cells derived from patients with BD. Preclinical data also showed that IFN‐γ was associated with social behavior, hippocampal plasticity, and cognition.^50^, ^51^ All these results suggested the correlation between NK cells and mood disorders. In our study, we showed significant differences in NK cells between BD and MDD. However, the pathophysiological mechanism of this phenomenon is unclear, and more basic studies are needed to investigate the underpinnings of these findings. Moreover, Kumar et al.^52^ found that T‐cell subsets of individuals of different ages were different. And in our study, we noticed that there were differences in age distribution among the three groups, which might be directly associated with the different onset ages of these two diseases. However, the bivariate analysis showed that the correlation between age and T‐cell subsets was relatively weak. Overall, the T‐cell proportion at variance among the three groups indicated different activated T‐cell immune statuses underlying MDD and alternative states of BD.

Based on the above findings, we further used peripheral proportions of T‐cell subsets and NK cells, and some significant demographic features to help distinguish between BD‐D and MDD patients. As a machine learning approach, a random forest model is a powerful tool for pattern recognition. Several studies using machine approaches have been conducted to provide help in clinical practice^53^; however, clinical translation is still a major challenge.^54^ Hence, our findings provided evidence for the potential of using T‐cell subsets as biomarkers to help distinguish between BD‐D and MDD patients.

Several limitations of this study need to be mentioned. First, this was a cross‐sectional, retrospective study, which precluded the establishment of firm causal inferences. Second, although the aim of this study was to investigate the differences in immune‐inflammatory profiles between MDD and BD, the lack of a group of healthy control appeared to be a major limitation. Third, the sample size of this study was relatively large when compared to previous studies,^16^, ^18^, ^55^ but the demographic characteristics such as age and BMI were not well controlled across the different groups. This also limits the further interpretation of our findings. Fourth, peripheral T‐cell subsets may not be stable under different physiological and pathological conditions. The data in our study were not repeatedly measured and could be inadequately convincing. In addition, although the AUC area of the model reached 0.77, there was still a 26.76% error rate in the auxiliary diagnosis of BD‐D and a 34.33% error rate in the diagnosis of MDD. Moreover, increasing evidence showed that psychotropic medications can affect the immune system.^56^ For example, lithium and valproate have been indicated to influence the decreased proliferative activity and increased susceptibility to apoptosis of T‐cell.^12^ The pharmacotherapy of depression also increases both the number and cytotoxic activity of NK cells.^57^, ^58^ In our study, there was no significant difference on the past use history of psychotropic medication among the three groups. However, we did not record the details of different psychotropic medicines, which may also limit the further interpretation of our findings.

In conclusion, to our knowledge, the present study has the largest sample size to explore the difference in T‐cell subsets between BD and MDD. Our findings suggest that T‐cell populations may be used as biomarkers to help distinguish BD from MDD, especially MDD and BD‐D. A random forest model may aid in the clinical differential diagnosis of BD‐D and MDD patients based on peripheral T‐cell populations and related demographic characteristics. Future studies with better designs are needed to verify our findings and unravel the underlying immune mechanisms.

## AUTHOR CONTRIBUTIONS

JL and SH designed the study. DL performed the statistical analyses. SL, DL, and CQ wrote the first draft of the manuscript. JJ, PZ, CX, LW, XG, YF, DZ, YC, HH, YZ, and XW interpreted the data and contributed to the manuscript with significant intellectual content. All authors have read and approved the final version of the manuscript prior to submission.

## FUNDING INFORMATION

This study was supported by grants from the Zhejiang Provincial Key Research and Development Program (Grant number: 2021C03107), the National Natural Science Foundation of China (Grant numbers: 81971271 and 82201676), the Zhejiang Provincial Natural Science Foundation (Grant number: LQ20H090013), and the Leading Talent of Scientific and Technological Innovation – Ten Thousand Talents Program of Zhejiang Province (Grant number: 2021R52016) and Innovation Group Program of Zhejiang Province (Grant number: 2020R01001).

## CONFLICT OF INTEREST STATEMENT

The authors have no conflict of interest to declare.

## Supporting information


Table S1.

## Data Availability

All data included in this study are available upon request by contact with the corresponding author.
