# Supplementary material for: Circulating T‐cell subsets discrepancy between bipolar disorder and major depressive disorder during mood episodes: A naturalistic, retrospective study of 1015 cases
Source: CNS Neurosci Ther. 2023 Jul 25;30(2):e14361. doi: 10.1111/cns.14361 (PMC10848094; doi:10.1111/cns.14361)
Supplement: Supplementary file 1 — Table S1. [file CNS-30-e14361-s001.docx]

**Supplementary materials**

Supplementary Table 1. Diagnostic criteria for BD-D, BD-M and MDD in *DSM-5*

Supplementary Table 2. Bivariate analysis result between T-cell subsets and age

Supplementary method. Measurement of T-cell surface markers

**Supplementary Table 1.** Diagnostic criteria for BD-D, BD-M and MDD in *DSM-5*

| Illness | Criteria |
| --- | --- |
| MDD | A: Five or more out of nine symptoms (including at least one of depressed mood and loss of interest or pleasure) for at least 2 consecutive weeks. Each of these symptoms represents a change from previous functioning.  B: Symptoms cause significant distress or impairment.  C: Episode not attributable to a substance or medical condition.  D: Episode not better explained by a psychotic disorder.  E: Absence of a manic or hypomanic episode.  Nine symptoms: 1. Depressed mood (subjective or observed); can be irritable mood in children and adolescents 2. Loss of interest or pleasure 3. Change in weight or appetite 4. Insomnia or hypersomnia 5. Psychomotor retardation or agitation (observed) 6. Loss of energy of fatigue 7. Worthlessness or guilt 8. Impaired concentration or indecisiveness 9. Thoughts of death or suicidal ideation or attempt. |
| BD-D | At least 1 essential criteria (depressed mood or anhedonia) persisting for ≥ 2 weeks and essential criteria and additional symptoms add up to ≥ 5 total diagnostic criteria being met.  Essential criteria: Persisting depressed mood; Persisting anhedonia  Additional symptoms criteria: Increase or decrease in appetite or body weight; Persisting insomnia or hypersomnia; Persisting fatigue or energy loss; Psychomotor agitation or slowing; Feelings of worthlessness or excessive guilt; Problems concentrating or making decisions; Recurring thoughts of death or suicide. |
| BD-M | At least 1 essential criterion persisting for 7 d (mania) or ≥ 4 d (hypomania) and  Essential criteria and additional symptoms add up ≥ 3 diagnostic criteria being met (≥ 4 if mood is irritable).  Assess for the threshold level of severity needed to distinguish between manic and hypomanic episodes.  Essential criteria: Persisting elevated, expansive, or irritable mood.  Additional symptoms criteria: Abnormally elevated self-esteem; Decreased need for sleep; Increased talkativeness; Fight of ideas or racing; Abnormal distractibility; Increased energy or goal-directed activity; Abnormally risky behaviors. |

**Abbreviations:** MDD: major depressive disorder; BD-M: bipolar disorder manic episode; BD-D: bipolar disorder depressive episode.

**Supplementary Table 2.** Bivariate analysis result between T-cell subsets and age

| Age |  | CD3+ | CD4+ | CD8+ | CD4+/CD8+ |
| --- | --- | --- | --- | --- | --- |
|  | R | -0.054 | 0.246 | -0.248 | -0.375 |
|  | *P* | 0.085 | < 0.001 | < 0.001 | < 0.001 |

**Supplementary Method.** Measurement of T-cell surface markers

Blood samples were stained with the following antibodies: Pcy5-conjugated anti-CD3, FITC-conjugated anti-CD4, P-phycoerythrin-conjugated anti-CD8 (BD Biosciences, CA, USA), and PE-conjugated anti-CD16/CD56 (Beckman Coulter, CA, USA). The frequency of different types of immunocompetent cells was characterized by flow cytometry. Briefly, at least 1 × 10^5^ cells were analyzed using BD FACS CantoTM II flow cytometer (BD Bioscience, CA, USA). The isotype-matched immunoglobulins were used as controls. The tests were performed at least in triplicate ^1^.

1. Wu W, Zheng Y-L, Tian L-P, et al. Circulating T lymphocyte subsets, cytokines, and immune checkpoint inhibitors in patients with bipolar II or major depression: a preliminary study. *Scientific reports.* 2017;7:40530.
